# Tangent space functional reconfigurations in individuals at risk for alcohol use disorder

**DOI:** 10.1162/netn_a_00419

**Published:** 2025-03-03

**Authors:** Mahdi Moghaddam, Mario Dzemidzic, Daniel Guerrero, Mintao Liu, Jonathan Alessi, Martin H. Plawecki, Jaroslaw Harezlak, David A. Kareken, Joaquín Goñi

**Affiliations:** Edwardson School of Industrial Engineering, Purdue University, West Lafayette, IN, USA; Purdue Institute for Integrative Neuroscience, Purdue University, West Lafayette, IN, USA; Department of Neurology, Indiana University School of Medicine, Indianapolis, IN, USA; Indiana Alcohol Research Center, Indiana University School of Medicine, Indianapolis, IN, USA; Department of Psychiatry, Indiana University School of Medicine, Indianapolis, IN, USA; Department of Epidemiology and Biostatistics, Indiana University Bloomington, Bloomington, IN, USA; Weldon School of Biomedical Engineering, Purdue University, West Lafayette, IN, USA

**Keywords:** Functional connectivity, Riemannian geometry, Regularization, Family history, Rest-task and task-rest fMRI

## Abstract

Human brain function dynamically adjusts to ever-changing stimuli from the external environment. Studies characterizing brain functional reconfiguration are, nevertheless, scarce. Here, we present a principled mathematical framework to quantify brain functional reconfiguration when engaging and disengaging from a stop signal task (SST). We apply tangent space projection (a Riemannian geometry mapping technique) to transform the functional connectomes (FCs) of 54 participants and quantify functional reconfiguration using the correlation distance of the resulting tangent-FCs. Our goal was to compare functional reconfigurations in individuals at risk for alcohol use disorder (AUD). We hypothesized that functional reconfigurations when transitioning to/from a task would be influenced by family history of AUD (FHA) and other AUD risk factors. Multilinear regression models showed that engaging and disengaging functional reconfiguration were associated with FHA and recent drinking. When *engaging* in the SST after a rest condition, functional reconfiguration was negatively associated with recent drinking, while functional reconfiguration when *disengaging* from the SST was negatively associated with FHA. In both models, several other factors contributed to the functional reconfiguration. This study demonstrates that tangent-FCs can characterize task-induced functional reconfiguration and that it is related to AUD risk.

## Author Summary

Human brain function constantly adapts to the external environment stimuli and transitions between cognitive states, which can be hindered by alcohol misuse and family history of alcohol use disorder (AUD). In this work, we used a novel methodology that applies Riemannian geometry concepts to functional connectivity to quantify functional reconfiguration of rest-to-task (engaging) and task-to-rest (disengaging) transitions. We ultimately aimed to determine the relationship between AUD risk factors and functional reconfiguration. Our findings showed that engaging functional reconfiguration was diminished in participants with a family history of AUD, whereas disengaging functional reconfiguration was diminished with greater recent drinking behavior. This study suggests that analysis of functional reconfiguration using Riemannian geometry is a promising avenue to better understand rest-to-task and task-to-rest brain transitions.

## INTRODUCTION

Brain functional connectivity has been used to predict behavioral attributes with the goal of understanding the relationship between individual characteristics (cognition, behavior, etc.) and brain functional networks (Amico et al., 2020; Eichenbaum et al., 2021; Fornito et al., 2015; Pervaiz et al., 2020; van der Wijk et al., 2022). These studies typically use data from fMRI scans that are acquired under one specific condition (e.g., during a quiet rest or while executing a motor or demanding attentional task).

A resting-state (task-free) functional magnetic resonance imaging (fMRI) design is now a default approach to assessing functional connectivity (Smitha et al., 2017), such as while participants fixate on a crosshair or closing their eyes, each without other specific cognitive demands. Functional connectomes (FCs) during rest have been used as predictors of various traits including intelligence (Finn et al., 2015; Hearne et al., 2016), attention (Rosenberg et al., 2016), impulsivity (Li et al., 2013), and cognitive deficits (Svaldi et al., 2021). They have also shown test-retest reliability in the form of functional connectivity *fingerprints* (Abbas et al., 2021, 2023; Amico & Goñi, 2018; Finn et al., 2015; Venkatesh et al., 2020), that is, as reflective of individual identity. The resting-state design is also well-suited for multisite (Bari et al., 2019) and longitudinal studies as it is independent of participants’ performance capabilities, attention span, age, or other specific limitations. (Finn et al., 2017; Rosenberg et al., 2016).

On the other hand, the resting state can also be considered as an unconstrained brain state, influenced by the level of wakefulness, affective state (e.g., mood, anxiousness, etc.), and other factors (Betzel et al., 2017; Buckner et al., 2013; Finn, 2021), including activities immediately preceding the resting-state scan (Chen et al., 2018; Tung et al., 2013). Others have therefore argued that functional connectivity from active cognitive engagement is a more controlled and specific probe that can better predict traits and behavior (Finn & Bandettini, 2021; Greene et al., 2018; Jiang et al., 2020; Zhao et al., 2023) and can be more easily interpreted (Finn, 2021).

Complementing both views, our group is interested in the “between states” functional reconfiguration involved in both the rise to task engagement and the task’s lingering aftereffects (Amico et al., 2020; Barnes et al., 2009; Chen et al., 2018). That is, transitions or functional reconfigurations between rest and task occur gradually and may vary across participants and clinical populations. Specifically, we are interested in brain endophenotypic risk from a family history of alcohol use disorder (AUD) and how it affects transitions to and from task demands. Those with a family history of AUD (FHA) have an approximate doubled risk for future AUD (Nurnberger et al., 2004). Insofar as FHA also affects executive cognitive ability (Braun et al., 2015; Gallen et al., 2016; Nurnberger et al., 2004; Schultz & Cole, 2016; Shine et al., 2019), it would seem likely that brain network flexibility would be affected.

Most FHA studies examine functional connectivity between a predefined region (a “seed”) and other areas, either at rest or during cognitive tasks (Cservenka, Casimo, et al., 2014; Cservenka, Fair, et al., 2014; Herting et al., 2011; Martz et al., 2019; Weiland et al., 2013; Wetherill et al., 2012). The findings are not entirely consistent but suggest greater ventral striatal-to-frontal functional connectivity in FHA (although the frontal regions vary) and less neocortical functional connectivity. Whole brain analyses of FHA with quantitative metrics that assess large-scale network organization across the whole brain are scarce. Two such network-level studies of adolescents found that frontal and premotor areas showed FHA-related changes in time-averaged resting-state functional connectivity that relate to impulsivity/externalizing symptoms and psychomotor speed (Holla et al., 2017; Vaidya et al., 2019). More recently, Elton et al. (2021) identified risk-related resting-state functional connectivity between pairwise nodes in healthy subjects whose siblings had either Diagnostic and Statistical Manual of Mental Disorders, Fourth Edition (DSM-IV) alcohol abuse or dependence, finding canonical correlations between (predominantly) frontoparietal nodes and cognitive and behavioral factors. Unusually, however, greater intelligence was related to the risk-associated connectivity differences in FHA.

In contrast, we previously studied functional connectivity immediately following a task in those with and without FHA, using connICA, an independent components analysis (ICA) framework (Amico et al., 2017), to dissect FCs into individual components of connectivity (Amico et al., 2020). It was found that individuals without FHA were featured most prominently in a particular functional connectivity component during the post-task rest period.

FCs are frequently represented as matrices computed using Pearson correlation coefficients between the time series of brain region pairs. FCs lie on a symmetric positive definite (SPD) manifold and are bound by its geometry (Dadi et al., 2019; You & Park, 2021). Therefore, their elements are inherently interrelated (You & Park, 2021), which may reduce the accuracy of predictions and associations (Tian & Zalesky, 2021) by violating “the uncorrelated feature assumption” (Ng et al., 2016). To overcome this problem, recent studies have instead proposed the use of tangent space projections of FCs (i.e., tangent-FCs) (Abbas et al., 2023; Pervaiz et al., 2020; You & Park, 2021), an application of Riemannian geometry (Pennec et al., 2006). The purpose of tangent space projection of FCs is to map the FCs to a Euclidean space, which removes the interrelatedness of functional couplings. This is accomplished by first computing a reference matrix, *C*_*ref*_, on the SPD manifold (Pervaiz et al., 2020). For every *C*_*ref*_, the derivatives of the curves crossing *C*_*ref*_ on the manifold form a Euclidean space that is tangent to *C*_*ref*_ (You & Park, 2021), that is, the tangent space of *C*_*ref*_. In this study, we computed *C*_*ref*_ as the Riemann mean of the FCs. Subsequently, we can compute tangent-FCs using the derivative of the curves that connect FCs to *C*_*ref*_ on the manifold. Tangent-FCs have been shown to be more reliable in finding associations with individual or demographic traits and conditions as compared with FCs (Dadi et al., 2019; Dodero et al., 2015; Ng et al., 2017; Qiu et al., 2015; Wong et al., 2018). More recently, Abbas et al. (2023) showed that tangent-FCs also carry a more precise fingerprint and Simeon et al. (2022) showed their application into harmonizing multisite data.

**Figure 1.**
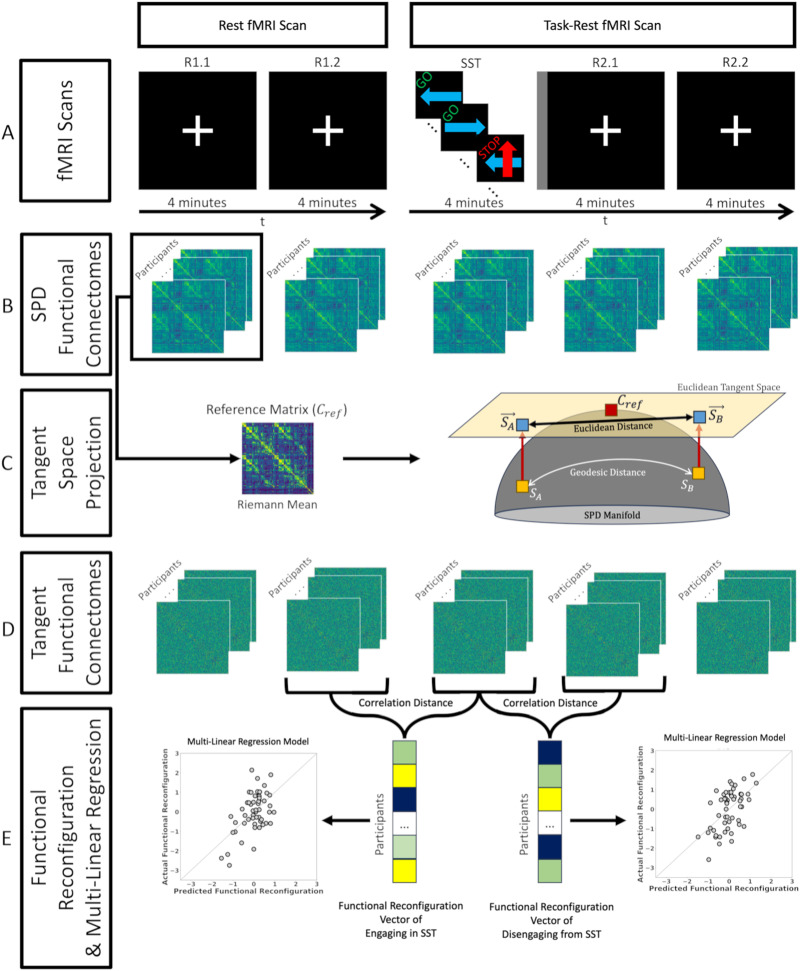
Study design and functional reconfiguration analysis scheme. (A) Each participant completed two fMRI scans. Scan 1 (left): 8 min of rest with eyes fixated on a central white crosshair. Scan 2 (right): 4-min SST (GO and STOP labels on top of each trial tile are only illustrative and did not appear on the actual stimuli). The SST segment of the scan was followed by a short 12-s intermission (*gray vertical stripe rectangle*) when a slide announced the upcoming rest with the printed statement, “The task is over. Fix your gaze on the crosshair for the remainder of the scan.” Participants then rested for 8 min and again fixated on the crosshair. (B) We divided Scans 1 and 2 into five 4-min segments (R1.1, R1.2, SST, R2.1, and R2.2) and estimated the functional connectivity between brain region pairs using Pearson’s correlation coefficient. We subsequently computed the Riemann mean of FCs from R1.1. (C) We used the reference computed in the previous step to project the FCs to tangent space, resulting in their tangent-FCs. (D) Functional reconfiguration of each participant from R1.2 to SST and SST to R2.1 were measured as the correlation distance of their corresponding tangent-FCs. We used these functional reconfiguration vectors in two multilinear regression models (engaging and disengaging), where the predictors included FHA, recent drinking behavior, and depressive symptoms (CES-D).

The aim of this work was to utilize tangent-FCs to determine the relationship between AUD risk factors and functional reconfiguration as individuals engage in and disengage from a task. Briefly, the computation of tangent-FCs requires (a) estimation of FCs, (b) regularization of FCs, and (c) computation of a reference matrix for tangent space projection. Our data were acquired in two consecutive fMRI scans. In the first scan, the participants underwent 8 min of rest, while in the second scan (immediately after), participants performed 4 min of SST followed by 8 min of rest. We estimated functional connectivity after dividing the two scans into five 4-min segments (Figure 1). We then regularized the resulting FCs and used the Riemann mean of the first resting-state segment as the reference matrix for tangent space projection (see the Riemannian Geometry in Functional Connectivity section for definitions). We then show that the choice of different resting-state fMRI segments to compute the reference minimally affects functional reconfiguration estimates (The Choice of fMRI Segment for Computing *C*_*ref*_ section). We also tested a range of regularization values and assessed their impact on the range and variance of tangent-FC elements that, in turn, can influence their predictive and explanation power (Regularization Stability section).

Finally, we used the correlation distance of tangent-FCs to measure functional reconfiguration from rest to task (engaging) and task to rest (disengaging). We hypothesized that task-to-rest and rest-to-task functional reconfiguration would be associated with AUD risk factors. We used multilinear regression analysis with engaging and disengaging functional reconfiguration as response variables to evaluate our hypothesis.

## MATERIALS AND METHODS

### Participant Information

The fMRI study data used here were previously reported by Amico et al. (2020) and consist of 54 participants (Table 1), among whom 23 were FHA positive, defined as having at least one first-degree relative with a history of AUD. FHA-negative participants had no first- or second-degree relatives with a history of AUD. We determined the family history through interviews using the family history module of the Semi-Structured Assessment for the Genetics of Alcoholism (Bucholz et al., 1994). All participants signed an informed consent prior to study procedures, all of which were approved by the Indiana University Institutional Review Board.

**Table 1.** Participant characteristics

	FHA positive (*n* = 23, 9 males)		FHA negative (*n* = 31, 16 males)		Full cohort (*n* = 54, 25 males)	
	Mean (*SD*)	Range	Mean (*SD*)	Range	Mean (*SD*)	Range
Age	23.04 (1.64)	21–26	22.35 (1.58)	21–26	22.64 (1.62)	21–26
Education	15.32 (1.22)	13–18	15.23 (1.20)	14–19	15.25 (1.20)	13–19
SSRT^a^ (ms)	250 (48)	157–397	230 (52)	124–346	238 (51)	124–397
CES-D^b^	8.04 (5.52)^d^	1–24	4.41 (4.21)^c^	0–17	5.96 (5.09)	0–24
AUDIT^d^	10.26 (6.52)	2–29	7.26 (3.92)	1–20	8.53 (5.35)	1–29
Drinking days	16.17 (8.02)	6–33	10.54 (5.60)	3–25	12.94 (7.24)	3–33
Drinks per week	12.27 (11.45)	2–51	7.11 (4.91)	1–21	9.31 (8.64)	1.20–51.40
Drinks per drinking day	3.66 (1.92)	1–9	3.67 (2.39)	1–10	3.67 (2.18)	1.30–10.40

^a^ Stop signal reaction time; time to withdraw a response.

^b^ Center for Epidemiologic Studies Depression scale. Scores of 16 or greater indicate a risk for clinical depression (Radloff, 1977).

^c^ Significant difference between FHA-positive and FHA-negative participants (*p* = 0.008).

^d^ Alcohol Use Disorder Identification Test (Saunders et al., 1993). A score of 8 or above suggests hazardous or harmful alcohol use.

Three FHA-positive participants had affected mothers but reported that their mothers did not drink during pregnancy. These three participants had 2 to 4 years of college education, had no obvious facial abnormalities as reported by the interviewing technicians, and had Go and Stop signal reaction times within one standard deviation of the remainder of the sample.

### Stop Signal Task

As described by Amico et al. (2020), the stop signal task (SST) consisted of 54 Go trials and 26 Stop trials, with brief practice (eight Go trials, seven stop trials) before imaging. Participants were instructed to respond as quickly and accurately as possible by pressing the right or left button on an MRI-compatible button box (Current Designs, Philadelphia, PA) that correspond to the Go signal of right- or left-pointing horizontal blue arrows. Stop trials (signaled by a red up-pointing arrow immediately following a Go stimulus) required participants to inhibit their Go response. An adaptive staircase algorithm adjusted the delay between the Go and Stop stimuli in 50-ms increments to help assure the 50% accuracy rate needed to calculate the stop signal reaction time (SSRT). This task was programmed using E-Prime 2.0 software (Psychology Software Tools Inc., Sharpsburg, PA). A participant’s estimated SSRT was calculated according to Band et al. (2003), by subtracting the average stop signal delay from that participant’s xth percentile Go response time, where x corresponds to the stop failure rate. Participants viewed stimuli back-projected on a screen at the rear of the scanner bore. Briefly, a lower SSRT reflects faster inhibition of a previously initiated response (Eagle et al., 2008). Participants’ performance during SST is presented in Table 2.

**Table 2.** SST performance

		SSRT (ms)	%Cor. Go	GoRT (ms)	DelayDur (ms)	Stop failure (%)
Full cohort (*n* = 54)	Average	238	0.95	454	190	49%
	*SD*	51	0.08	91	99	7%
FHA positive (*n* = 23)	Average	250	0.94	453	177	50%
	*SD*	48	0.08	88	94	7%
FHA negative (*n* = 31)	Average	230	0.95	455	199	48%
	*SD*	52	0.09	95	103	7%

%Cor. Go = percent Go correct; GoRT = Go reaction time; DelayDur = average time between Go and Stop stimulus; and Stop failure = percentage of stop trials on which subjects made a Go response.

### MRI Acquisition and Preprocessing

Participants were imaged in a 3 T Siemens Prisma MRI scanner with a 64-channel head coil (neck elements off). fMRI data were acquired with a blood oxygenation level dependent (BOLD) contrast-sensitive sequence (gradient-echo echo planar imaging (GE-EPI), multiband slice acceleration factor 3, repetition/echo time TR/TE = 1,200 ms/29 ms, flip angle of 65°, voxels of 2.5 × 2.5 × 2.5 mm^3^, field of view of 220 × 220 mm^2^, 54 interleaved axial slices). During the first BOLD fMRI scan (8:00 min; 400 volumes) participants were at rest and instructed to fixate their gaze on a central white crosshair. The next fMRI scan (12:12 min, 610 volumes) included 4 min of SST performance, followed by a short 12-s transition period when a slide announced an upcoming 8-min rest (Figure 1A). The initial 7 s in each scan (calibrations; magnetic resonance signal reaching steady-state magnetization) was excluded from subsequent analyses.

Participants first received a whole-brain T1-weighted structural MRI (3D magnetization prepared rapid gradient echo (MPRAGE) sequence; duration of 5:12 min, 176 sagittal slices, voxels of 1.1 × 1.1 × 1.2 mm^3^, generalized autocalibrating partially parallel acquisition *R* = 2 acceleration). In addition, two short (16 s) spin echo EPI scans (TR/TE = 1,560/49.8 ms, five in the A-P and five in the P-A direction, same imaging volume/voxel size as for the GE-EPI scans) were acquired immediately before each BOLD fMRI scan. These phase-reversed spin echo EPI scans provided field maps to correct EPI geometric distortion using FMRIB Software Library’s (FSL’s) topup/applytopup (Smith et al., 2004).

The analysis pipeline originally applied to this dataset by Amico et al. (2020) was updated to include the state-of-the-art preprocessing techniques (e.g., BOLD data denoising, single-step regression) and newer software versions (Halcomb et al., 2024). The preprocessing was implemented with in-house bash scripts using Python 3.6 and (version 6.0.1). The T1-weighted MPRAGE image of each participant was denoised prior to brain masking and extraction with ANTs (Avants et al., 2009) and then nonlinearly transformed (FSL’s flirt and fnirt) to Montreal Neurological Institute (MNI) MNI152 standard space. This MNI-to-T1 transformation was followed by T1-to-EPI transformation, which included linear, nonlinear, and boundary-based registration (Greve & Fischl, 2009) in FSL. This procedure improves registration between the structural masks and functional data and generates standard-to-native (MNI-to-EPI) and inverse (EPI-to-MNI) space transformations required to apply standard space atlases in native EPI space. Functional connectivity data were processed in the native EPI space of each participant. The preprocessing included BOLD volume distortion correction using FSL’s topup/applytopup (utilizing phase-reversed spin echo field mapping scans), head motion realignment (mcflirt), earlier described T1-to-EPI registration, normalization to mode 1000, and spatial smoothing with a 6-mm isotropic full width at half maximum full width at half maximum Gaussian kernel.

Following recommendations for robust preprocessing (Eklund et al., 2016), the preprocessed data were entered into FSL’s MELODIC (Nickerson et al., 2017) for ICA-based denoising with ICA-AROMA (Pruim, Mennes, Buitelaar, et al., 2015; Pruim, Mennes, van Rooij, et al., 2015). A single-step regression was applied to the denoised BOLD volumes to avoid reintroducing artifacts in the preprocessed denoised data (Lindquist et al., 2019; Phạm et al., 2023). Specifically, regressors were applied that indexed head motion (from the realignment and their derivatives; Power et al., 2014) accounted for physiological noise (first five signals obtained by principal component analysis from the white matter and cerebrospinal fluid-eroded masks; an implementation of aCompCor; Muschelli et al., 2014) and three components of the global signal (mean, derivative, squared). Additional regressors performed high-pass filtering (fmin = 0.009 Hz) using discrete cosine transform bases (Shirer et al., 2015) and included outlier volume despiking (Phạm et al., 2023). The outlier volumes were scrubbed based on the significant “DVARS” metrics obtained on the single-regression preprocessed data as described in Phạm et al. (2023). The scrubbed volume values were replaced using the linear interpolation of their nearest remaining time points. The mean number of the scrubbed volumes was 0.5% (standard deviation: 0.35%; range: 0–1.5%) across both scans for all participants. The number of scrubbed volumes between the two FHA groups did not differ (*p* = 0.20 for the first resting-only fMRI scan, *p* = 0.79 for the second, task-rest scan; two-tailed *t* test).

A whole-brain data-driven functional parcellation based on 300 regions, as obtained by Schaefer et al. (2018), was projected into a native EPI space of each participant as described earlier. For the subcortex, we used a 32-node Scale II parcellation (Melbourne subcortical atlas) as defined by Tian et al. (2020), which resulted in 332 total brain regions.

### Riemannian Geometry in Functional Connectivity

Functional connectivity is most often estimated pairwise using the Pearson correlation coefficient of two brain regions’ BOLD time series, resulting in a symmetric correlation matrix for whole-brain functional connectivity (Fornito et al., 2016). Such symmetric matrices are positive definite if they are invertible (all their eigenvalues are greater than 0). The collection of such matrices forms a nonlinear topological space, or manifold, that is referred to as SPD (You & Park, 2021). If correlation matrices are singular (at least one eigenvalue equal to 0), they are positive semidefinite and lie on the edge of the SPD manifold. This occurs, for instance, when computing FCs if the parcellation has more brain regions than the number of time points in the BOLD time series. Such matrices, however, become positive definite through regularization, where their main diagonal entries are incremented by a regularization value (Abbas et al., 2021; Venkatesh et al., 2020).

The canonical methods that use Euclidean or correlation distance of vectorized FCs ignore their topological properties and the interrelatedness between their elements (Abbas et al., 2023; You & Park, 2021). Consequently, comparisons of FCs belonging to the SPD manifold must account for their non-Euclidean geometry by using the Affine Invariant Riemannian Metric (AIRM) (Pennec et al., 2006), also known as geodesic distance. Alternatively, we can apply tangent space projection, where the FCs are projected into a Euclidean space that is tangent to a reference matrix, *C*_*ref*_, on the SPD manifold (Pervaiz et al., 2020). For any reference matrix belonging to the SPD manifold, its tangent space is a collection of vectors that are the derivatives of the curves crossing that matrix on the manifold (You & Park, 2021). The geodesic distance between FCs on the manifold can be approximated by the Euclidean distance of their corresponding projections on the tangent space (Barachant et al., 2013). The projection is computed using the formula in Equation 1.

$$\hat{S}=\log_{m}\left(C_{ref}^{-\frac{1}{2}}\cdot S\cdot C_{ref}^{-\frac{1}{2}}\right)$$ (1)

where $\hat{S}$ is the projected matrix on tangent space, *S* is the matrix on the SPD manifold, *C*_*ref*_ is the reference matrix on the manifold, and log_*m*_ is the matrix logarithm function.

Estimated FCs are always positive semidefinite. However, they are not guaranteed to be positive definite and, hence, may not be invertible. In that case, main diagonal regularization is an essential step when using geodesic distance or tangent space projection. The choice of regularization value can affect the resulting tangent-FCs, thus impacting fingerprinting accuracy (Abbas et al., 2023) and predictions based on tangent-FCs. The reference matrix choice, *C*_*ref*_, can also affect the outcome. The reference matrix, which represents a centroid of the data, is often obtained as a function of all or a subset of FCs in the dataset (Pervaiz et al., 2020).

Abbas et al. (2023) evaluated manifold and tangent-FCs fingerprinting accuracy (Amico & Goñi, 2018; Finn et al., 2015)—an important criterion for the reliability of FCs and the predictions or models based on them. They found that (a) the combination of correlation distance and tangent-FCs had the highest fingerprinting accuracy for all Human Connectome Project fMRI conditions and for all parcellation granularities evaluated; (b) the optimal regularization value for fingerprinting (Abbas et al., 2021) was consistently 0.01 (the smallest value tested) for the combination of correlation distance and tangent-FCs; and (c) the Riemann function, defined in Equation 2 (Fletcher et al., 2004; Moakher, 2005), was the best choice to compute the reference, *C*_*ref*_, for fingerprinting accuracy.

$$C_{ref}=\arg\;\min_{C}\sum_{i}d_{G}{\left(\hat{C_{ref}},S_{i}\right)}^{2}$$ (2)

where *C*_*ref*_ is the Riemann mean reference matrix, *S*_*i*_ is the *i*th SPD matrix, $\hat{C_{ref}}$ is the initial matrix for the computation of *C*_*ref*_ (e.g., arithmetic mean of *S*_*i*_, ∀*i*; Pervaiz et al., 2020), and *d*_*G*_ is the geodesic distance function. After projection, the elements of the projected matrices become unrelated features (Ng et al., 2016). We can thus use correlation distance to compare tangent-FCs.

### Functional Reconfiguration Analysis

We estimated the pairwise functional connectivity of brain regions by calculating the Pearson correlation coefficient of their corresponding preprocessed BOLD time series. Both scans were divided into 4-min segments (the SST duration), resulting in one SST segment and four resting-state segments (R) and labeled as R1.1, R1.2, SST, R2.1, and R2.2 (Figure 1). Regularized FCs were projected to tangent space using the Riemann mean of R1.1 FCs as the reference using PyRiemann library (Barachant, 2015). We defined the functional reconfiguration from changing task demands (from rest-to-task or from task-to-rest) as the distance between their corresponding FCs (SPD FCs or tangent-FCs). Specifically, we measured the functional reconfiguration of each participant from R1.2 to SST (engaging the task) and SST to R2.1 (disengaging from the task) by calculating the correlation distance of their corresponding tangent-FCs (Figure 1E).

The engaging and disengaging functional reconfiguration of participants served as the response variable in a multilinear regression model, with sex, age, education, SSRT, Center for Epidemiologic Studies Depression scale (CES-D) score, recent drinking behavior, and FHA status (0 = negative, 1 = positive) as predictors. Here, the recent drinking behavior was derived as the first principal component of the Alcohol Use Disorder Identification Test (AUDIT) score, as well as self-reported drinking days, drinks per week, and drinks per drinking day (derived from the timeline follow-back technique; Sobell et al., 1986), which explained 65% of variance. We performed two separate leave-one-out cross validations to evaluate the stability of the final models (regression coefficients) with respect to (a) the sample variation in the final models and (b) the sample variation in computing the reference, *C*_*ref*_, for tangent space projection. We also tested a range of regularization values (0.001–10) to assess their effects on the estimation of functional reconfiguration and ultimately the multilinear regression models.

## RESULTS

### The Choice of fMRI Segment for Computing *C*_*ref*_

**Figure 2.**
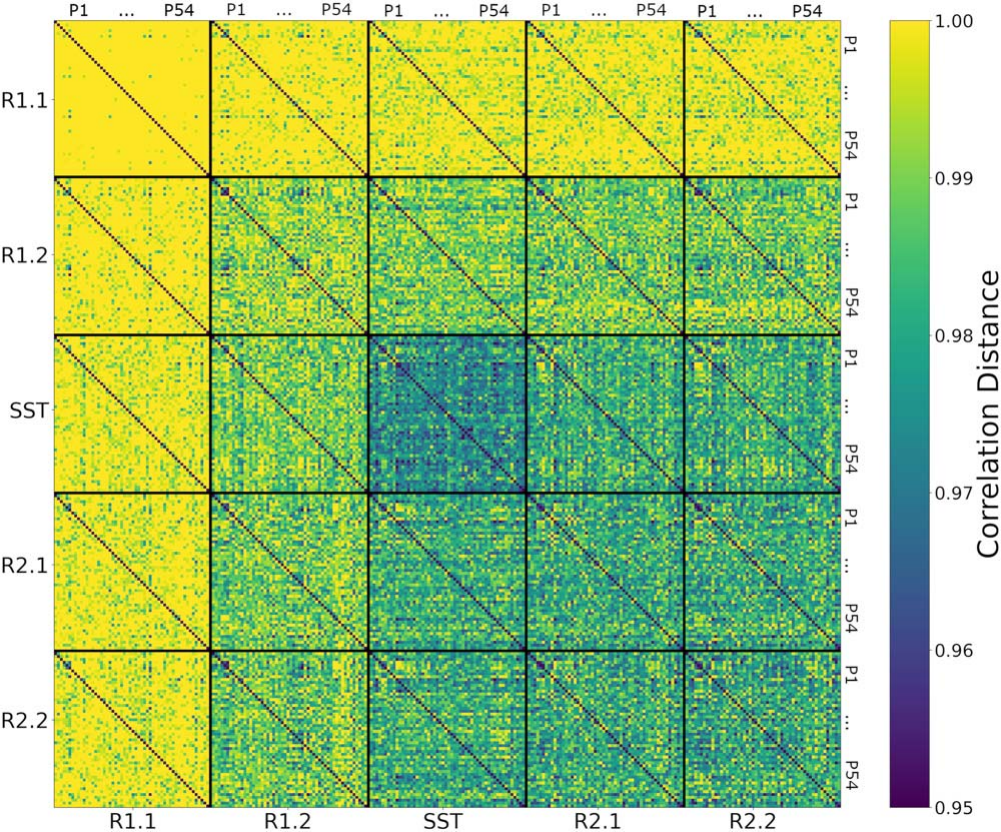
The meta-identifiability matrix for five fMRI segments consists of 25 identifiability matrices. Each of the five within-segment identifiability matrices is symmetric, contains correlation distances of tangent-FCs across all 54 participants (denoted as P1 … P54), and has zeros on its main diagonal (zero distance between a tangent-FC and itself). Note that identifiability matrices involving different segments are not symmetric. Furthermore, they not only show between-participants distances but also contain, on their main diagonal, functional reconfiguration of each participant from one fMRI segment to another.

After computing the correlation distance of tangent-FCs across all participants for a pair of fMRI segments (e.g., R1.1 and R1.2), we can represent the result as a matrix of size 54 × 54, that is, an *identifiability matrix* (Amico & Goñi, 2018). The diagonal elements of this matrix represent the functional reconfiguration of each participant. We computed the identifiability matrices of all pairwise comparisons across fMRI segments (25 matrices) and stacked them to produce a *meta-identifiability matrix* (Figure 2).

As shown in Figure 2, the off-diagonal values of identifiability matrices that involve R1.1 are noticeably larger due to the use of R1.1 manifold FCs in the computation of the reference *C*_*ref*_ for tangent space projection. This is because the commonalities of the FCs that are used to compute the reference are cancelled out after projection, hence, the large correlation distance between them. This has been described as data *whitening* with respect to the reference in the Riemannian geometry literature (Dadi et al., 2019; Varoquaux et al., 2010).

**Figure 3.**
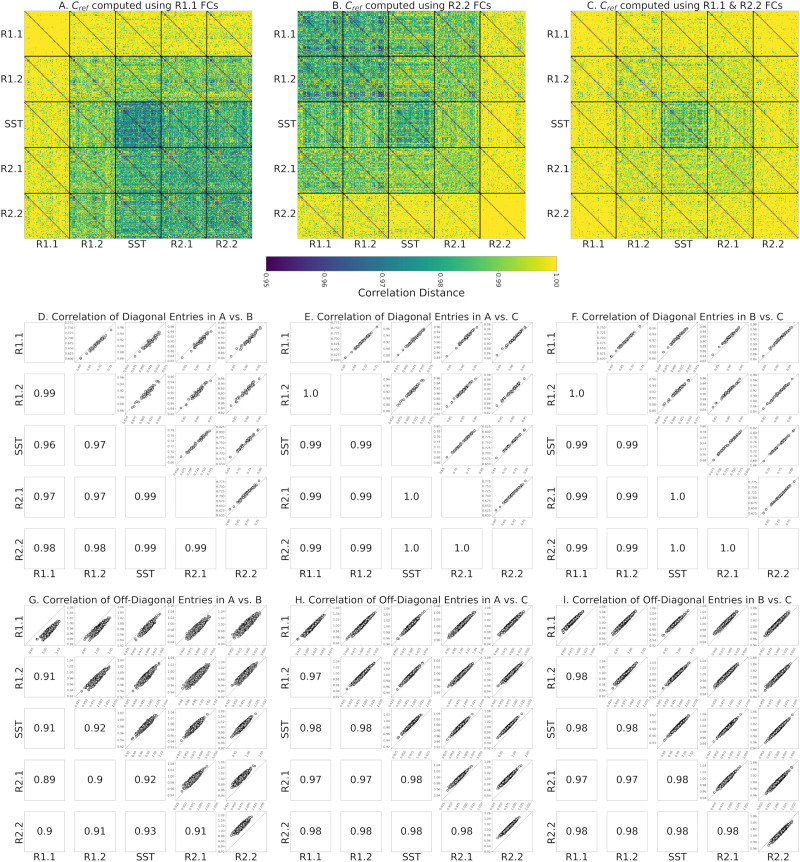
(A) Meta-identifiability matrix of tangent-FCs with *C*_*ref*_ computed using R1.1 FCs. (B) Meta-identifiability matrix of tangent-FCs with *C*_*ref*_ computed using R2.2 FCs. (C) Meta-identifiability matrix of tangent-FCs with *C*_*ref*_ computed using R1.1 and R2.2 FCs. The color bar for (A), (B), and (C) is the same as Figure 2. (D) Correlation of diagonal entries of identifiability matrices that belong to the meta-identifiability matrices in (A) and (B). (E) Correlation of diagonal entries of identifiability matrices that belong to the meta-identifiability matrices in (A) and (C). (F) Correlation of diagonal entries of identifiability matrices that belong to the meta-identifiability matrices in (B) and (C). (G) Correlation of the off-diagonal entries of identifiability matrices that belong to the meta-identifiability matrices in (A) and (B). The correlations on the main diagonal are 0.90, 0.91, 0.95, 0.90, and 0.92. (H) Correlation of the diagonal entries of identifiability matrices that belong to the meta-identifiability matrices in (A) and (C). Correlations on the main diagonal are 0.97, 0.98, 0.99, 0.97, and 0.98. (I) Correlation of the diagonal entries of identifiability matrices that belong to the meta-identifiability matrices in (B) and (C). Correlations on the main diagonal are 0.98, 0.98, 0.99, 0.97, and 0.98.

The same phenomenon appears when using the Riemann mean of R2.2 FCs or the Riemann mean of both R1.1 and R2.2 FCs as the reference matrix, *C*_*ref*_, for tangent space projection (Figures 3B and 3C, respectively, compared with Figure 3A). Therefore, we evaluated how the use of different resting-state fMRI segments to compute the reference, *C*_*ref*_, impacts the estimation of within-participant functional reconfiguration (**diagonal elements** of identifiability matrices) and between-participants distances (**off-diagonal elements** of identifiability matrices). Specifically, we separately computed the correlation of diagonal elements (Figures 3D–F) and off-diagonal elements (Figures 3G–I) of each identifiability matrix across pairs of references. Overall, these results indicate that the choice of nontransitioning resting-state fMRI segment to compute *C*_*ref*_ minimally affects the estimation of the functional reconfiguration values. As shown in Figures 3D–E, the correlation of functional reconfiguration estimates (diagonal elements of each identifiability matrix) using the Riemann mean of R1.1 as the reference and the two alternatives always exceeds 0.96. Note that to preserve the intrinsic characteristics of SST in the tangent-FCs, we considered only resting-state fMRI segments when computing the reference.

### Identification Rates

**Figure 4.**
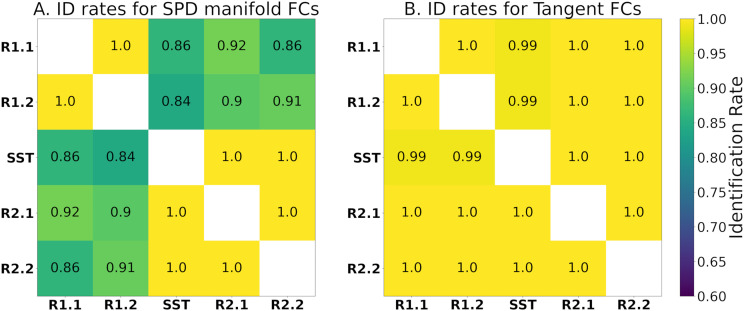
Identification rates of participants across fMRI segments with (A) SPD manifold FCs and (B) tangent-FCs (regularization value of 0.001 and reference computed using R1.1 FCs). Note that the tangent space projection removes potential scan effects in terms of fingerprinting.

In a recent study (Abbas et al., 2023), tangent space projection of FCs improved fingerprinting (measured by identification rate) for all evaluated fMRI conditions and parcellation granularities. The identification rates of this dataset within the blocks of the meta-identifiability matrix are presented before and after tangent space projection, that is, on the SPD manifold FCs (Figure 4A) and for tangent-FCs (Figure 4B). Near-perfect identification rates across two scans only occur with tangent-FCs. This suggests that tangent space projection is mitigating biases due to scan effects.

### Functional Reconfiguration

**Figure 5.**
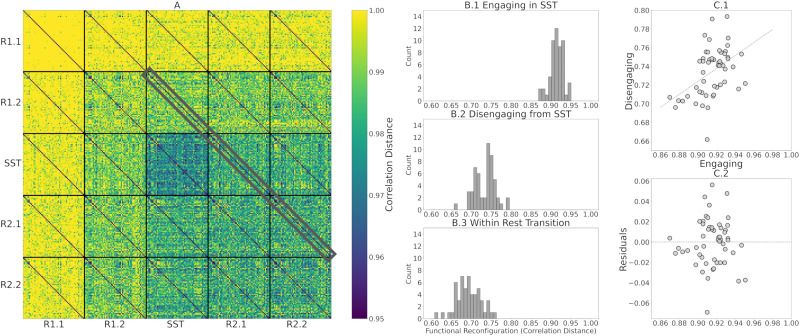
(A) Meta-identifiability matrix with functional reconfiguration vectors of R1.2 to SST (engaging), SST to R2.1 (disengaging), and R2.1 to R2.2 (within-rest) highlighted in gray. (B.1 to B.3) Histograms of functional reconfiguration values of engaging in the SST, disengaging from the SST, and within-rest post-SST, respectively. We tested functional reconfiguration using paired t tests among engaging, disengaging, and within-rest models and observed significant differences (*p* < 0.001). (C.1) Scatterplot of engaging versus disengaging functional reconfiguration vectors, showing that the two are unrelated (*r* = 0.44). (C.2) Scatterplot of residuals when predicting disengaging from engaging.

Functional reconfiguration from rest to task (R1.2 to SST), task to rest (SST to R2.1), and rest to rest (R2.1 to R2.2, as a baseline control comparison within rest) are highlighted in Figure 5A. The histograms of functional reconfiguration vectors in Figures 5B.1 to 5B.3 show that individuals functionally reconfigure more when engaging in the SST compared with disengaging from it. Furthermore, the correlation between functional reconfiguration vectors of engaging in and disengaging from the SST is 0.45 (Figure 5C), suggesting that engaging and disengaging from SST for each participant are only partially related. We, therefore, analyzed engaging and disengaging functional reconfiguration with separate multilinear regression models to assess the AUD risk factors associated with each transition (see the Associations Between Functional Reconfiguration and AUD Risk section).

### Associations Between Functional Reconfiguration and AUD Risk

We performed three multilinear regression analyses (engaging, disengaging, and within rest) to determine how functional reconfiguration is related to AUD risk. We accounted for possible confounding nuisance variables (age; sex coded as male 1, female 0; education; SSRT on which the FHA groups differed) and related AUD risk variables on which the FHA groups also differed (recent drinking, negative affect/depressive symptoms, assessed by CES-D scores). We first assessed, iteratively, how the cumulative explained variance of the model evolved as the predictors that were added in the order described above. This approach showed the contribution of each predictor to the existing model. Subsequently, we built the final models with all seven predictors. Note that the order of the predictors does not affect the results of the final models. The response variable in each model is the functional reconfiguration vector of (a) *engaging in the SST*, that is, transition from rest to task (R1.2 to SST); (b) *disengaging from the SST*, that is, transition from task to rest (SST to R2.1); and (c) *within-rest* transition (R2.1 to R2.2, following the SST) as an intended comparator in which there should be no systematic engagement or disengagement.

**Figure 6.**
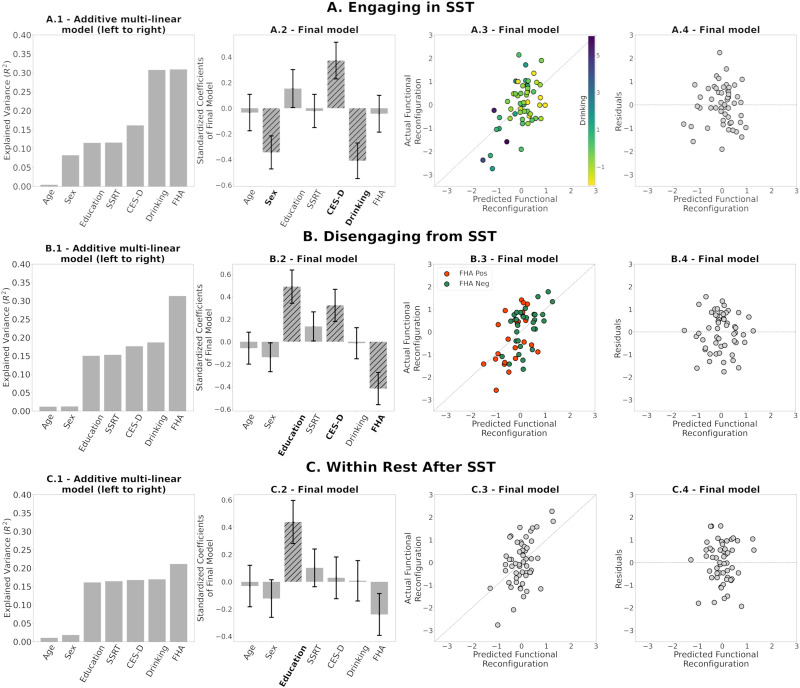
(A.1, B.1, C.1) Additive multilinear regression of AUD-risk variables (and adjustment covariates) on functional reconfiguration of engaging in the task (R1.2 to SST), disengaging from the task (SST to R2.1), and during rest after SST (R2.1 to R2.2), with predictors sequentially introduced in the order depicted. See the Materials and Methods section for variable definitions. (A.2, B.2, C.2) Coefficients of the final multilinear regression model, including standard error. Hatched bars and bold labels denote significant predictors (*p* ≤ 0.05). (A.3, B.3, C.3) Scatterplots of predicted versus actual functional reconfiguration values for the final multilinear regression model in (A1), (B1), and (C1), respectively. Colors in (A.3) and (B.3) are based on (standardized) recent drinking score and FHA, respectively. (A.4, B.4, C.4) Scatterplots of the predicted functional reconfiguration versus the standardized residuals of each participant for the final models in (A.1), (B.1), and (C.1), respectively.

When examining the functional reconfiguration of *engaging in the SST*, the model explained 31% of the variance (Figures 6A.1–A.4). Significant predictors were sex (*p* = 0.01), CES-D (*p* = 0.012), and recent drinking (*p* = 0.005). Being male and having higher recent drinking correspond with smaller engaging functional reconfiguration, whereas a higher CES-D score corresponds with greater engaging functional reconfiguration.

Similarly, when examining the functional reconfiguration of *disengaging from the SST*, the model explained 31% of the variance (Figures 6B.1–B.4). Significant predictors were education (*p* = 0.002), CES-D (*p* = 0.028), and FHA (*p* = 0.005). Higher education and CES-D score correspond with greater disengaging functional reconfiguration, while being FHA positive corresponds with smaller disengaging functional reconfiguration.

Finally, the model for the functional reconfiguration of *within-rest transition* explained 21% of the variance (after SST; Figure 6C) and 18% of the variance (before SST; Supporting Information Figure S2A). Note that these two models have less explained variance than engaging and disengaging models. Furthermore, within-rest transitions have significantly smaller magnitudes of functional reconfiguration (Figure 5). The only significant predictor in both models was education (*p* = 0.009 and *p* = 0.016, respectively), with higher education corresponding with greater within-rest functional reconfiguration before and after SST. None of the AUD risk-related variables were significant.

Residual diagnostics for all three models indicate that the linear regression assumptions were not violated; see the residual plots in Figures 6A.4, 6B.4, and 6C.4. Finally, when adding a head motion (indexed by number of scrubbed volumes across the two scans), it did not change the significance of the other predictors in the multilinear models, explained little variance by itself, and was not a significant predictor in any of the final models (Supporting Information Figure S3).

### Assessing the Stability of Regression Coefficients of Final Models

**Figure 7.**
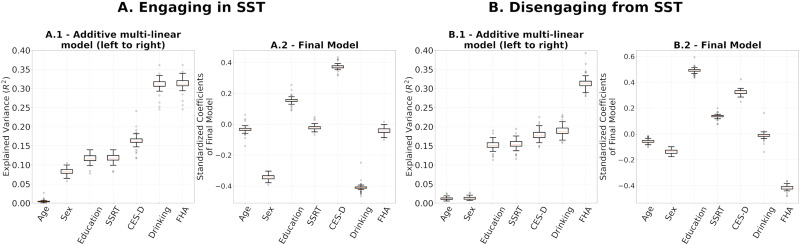
(A.1, B.1) Leave-one-out additive multilinear regression model explaining engaging and disengaging functional reconfiguration, respectively. (A.2, B.2) Distributions of leave-one-out coefficients.

**Figure 8.**
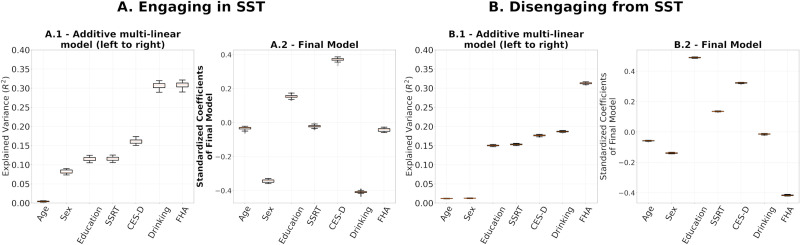
(A.1, B.1) Full additive multilinear regression models explaining engaging and disengaging functional reconfiguration, respectively, with the reference for tangent space projection, *C*_*ref*_, built based on leave-one-out coefficients. (A.2, B.2) Distributions of leave-one-out coefficients.

To evaluate final models’ stability, we performed leave-one-out cross validations for (a) the multilinear models after using the entire set of SPD FCs of R1.1 to build the reference and project the FCs to tangent space (Figure 7) and (b) the computation of the reference for tangent space projection. In the latter, we project the SPD FCs to tangent space 54 times, each with *C*_*ref*_ built on 53 FCs, leaving one participant out. On each iteration, we built a multilinear regression model for engaging in and disengaging from the SST on the projected FCs, which included all 54 participants (Figure 8). The rationale was to understand how much variance in our results comes from the data used for the multilinear models versus the data used to build the reference. The results are presented in Figures 7 and 8, respectively.

### Regularization Stability

**Figure 9.**
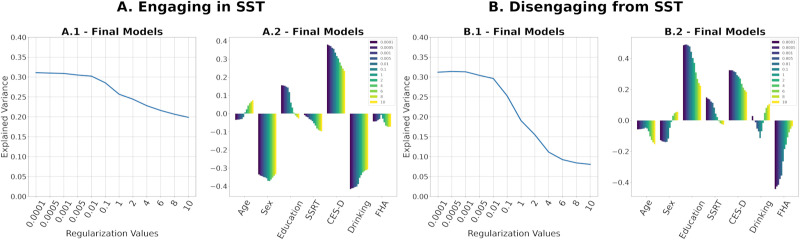
(A.1, B.1) Explained variance of multilinear regression models built on tangent-FCs that were projected to tangent space after the regularization of SPD FCs with the regularization values on the *x*-axis. (A.2, B.2) Coefficients of the multilinear regression models, each ordered by the regularization value of their corresponding tangent-FCs.

Regularization is essential to ensure the positive definiteness of SPD FCs for tangent space projection (see the Riemannian Geometry in Functional Connectivity section for details). It is noteworthy that the magnitude of regularization value has an inverse relationship with the range of tangent-FC elements. Moreover, two tangent-FCs derived from the same FC but based on two different regularization values are not guaranteed to be highly correlated (Abbas et al., 2023). This can consequently influence the estimation of functional reconfiguration. Therefore, we tested the performance of the multilinear models presented in the Associations Between Functional Reconfiguration and AUD Risk section across a range of regularization values (0.0001–10). Figure 9 shows that the multilinear regression models explain more variance of functional reconfiguration for regularization values ≤0.01. Furthermore, regularization values also affect the significance of the predictors as shown in Table 3.

**Table 3.** Effect of regularization on the significant predictors of the final engaging and disengaging models (regularization value = 0.001; shown in bold); for every regularization value, tangent-FCs were computed, and functional reconfiguration models were obtained using all seven predictors (see Figure 9 for the effect on regression coefficients)

Regularization values	A. Engaging in the SST			B. Disengaging from the SST		
	*p* Values					
	Sex	CES-D	Drinking	Education	CES-D	FHA
0.0001*	0.012	0.010	0.004	0.002	0.027	0.003
0.0005*	0.010	0.011	0.005	0.002	0.027	0.004
**0.001** *	**0.010**	**0.012**	**0.005**	**0.002**	**0.028**	**0.005**
0.005*	0.009	0.015	0.006	0.002	0.032	0.011
0.01*	0.009	0.016	0.006	0.002	0.036	0.017
0.1	0.009	0.025	0.008	0.006	0.055	0.082
1	0.007	0.038	0.017	0.015	0.073	0.237
2	0.008	0.048	0.023	0.028	0.094	0.327
4	0.010	0.069	0.031	0.071	0.151	0.502
6	0.013	0.090	0.037	0.121	0.203	0.641
8	0.016	0.112	0.041	0.164	0.241	0.744
10	0.020	0.133	0.044	0.196	0.267	0.815

* Regularization values for which all *p* ≤ 0.05.

When assessing the relationship between the regularization value and the variance of tangent-FC elements in our dataset, we discovered that for regularization values beyond 0.1, the range and variance of tangent-FC elements become very small, possibly limiting the explanation capacity of our models (Supporting Information Figure S4).

## DISCUSSION

In this work, we evaluated the relationship between AUD risk factors and functional reconfiguration both in and from the SST of motor inhibition. We applied a new methodology to quantify rest-to-task (R1.2 to SST) and task-to-rest (SST to R2.1) functional reconfigurations by calculating the correlation distance of the corresponding tangent-FCs for each participant. Tangent-FCs are the result of applying a Riemannian geometry approach, that is, tangent space projection (Pennec et al., 2006) to FCs, which projects them to a Euclidean space and transforms their elements into independent features (Abbas et al., 2023; Pervaiz et al., 2020; Tian & Zalesky, 2021; You & Park, 2021). This approach overcomes the limitations of using SPD manifold FCs, specifically interrelatedness of the elements. We also introduced the concept of a meta-identifiability matrix (Figure 2). Given that we divided the scans into five fMRI segments, this symmetric matrix is composed of 25 identifiability matrices. The matrices on the main diagonal correspond with the within fMRI segment comparisons, whereas the remaining matrices are comparisons across pairs of fMRI segments. The meta-identifiability matrix depicts the relationship between fMRI segments and participants.

As illustrated in Figures 3A–C, the choice of fMRI segment for computing the reference for tangent space projection affects the magnitude of off-diagonal entries (correlation distance of tangent-FCs across participants) in the meta-identifiability matrix. Specifically, by applying tangent space projection, we remove the commonalities of the FCs that are used in the computation of the reference matrix. However, our analysis shows that functional reconfiguration vectors (diagonal elements of each identifiability matrix) across the three meta-identifiability matrices in Figures 3A–C are highly correlated (≥0.96) (Figures 3D–F), and despite the difference in the magnitude of off-diagonal elements, they are also highly correlated across the three computations of the reference matrix (≥0.89) (Figures 3G–I). Functional reconfiguration estimates are thus stable with respect to the fMRI segments used to compute the reference (Riemann mean of all FCs in that segment).

Recent studies show that tangent space projection improves the predictive power of FCs (Dadi et al., 2019; Dodero et al., 2015; Ng et al., 2017; Qiu et al., 2015; Wong et al., 2018) and fingerprinting accuracy (Abbas et al., 2023), which is also evident in our results (Figure 4). Tangent space projection improved identification rates between segments that belong to different fMRI scans, which suggests that scan effects were minimized. This result comports with recent studies showing how tangent-FCs can help harmonize multisite data (Simeon et al., 2022). This is a key regarding our measurements of functional reconfiguration, particularly engaging in the SST, as the transition between rest and task occurred across two scans.

As illustrated in Figure 5B, individuals exhibited more functional reconfiguration when engaging in the SST compared with disengaging from it, both of which are significantly greater than the functional reconfiguration of within-rest transition (R2.1 to R2.2) (*p* < 0.001). The difference in the magnitude of functional reconfiguration between engaging in and disengaging from the SST is consistent with previous findings about lingering task effects in subsequent rest periods (Barnes et al., 2009; Chen et al., 2018).

As AUD is highly prevalent (Grant et al., 2015), understanding brain-related vulnerabilities is important to the prevention, treatment, and, more generally, public health, especially given AUD’s comorbidity and joint risk with other mental illness (Walters et al., 2018). Prior research of how FHA affects brain connectivity is not extensive, with past work using a priori seed regions (Cservenka, Casimo, et al., 2014) or seed-based analyses of data collected during cognitive tasks (Herting et al., 2011; Weiland et al., 2013; Wetherill et al., 2012). These data suggest that FHA may affect the functional connectivity of the reward and frontal circuits, as evident from related work (Cservenka, 2016). Broader analyses of the whole-brain regional network connectivity across the brain from resting-state studies are not only less common but also suggest altered frontal and dorsal premotor and sensorimotor connectivity between those with and without FHA (Holla et al., 2017; Vaidya et al., 2019).

Functional reconfiguration can be measured at multiple spatial and temporal scales within a variety of frameworks, such as similarity measures of FCs (Schultz & Cole, 2016), whole-brain network structure (Shine & Poldrack, 2018), short-term fluctuations of whole-brain FCs (Gonzalez-Castillo et al., 2015), trapping efficiency and exit entropy of functional networks (Duong-Tran et al., 2021), and modularity and community detection (Bassett et al., 2011; Betzel et al., 2017). Conceptually, functional reconfiguration can be interpreted as relating to (a) *efficiency*, wherein less functional reconfiguration indicates more efficient transitions between task and rest (Duong-Tran et al., 2021; Schultz & Cole, 2016), and (b) *flexibility*, wherein functional reconfiguration is commensurate to the required behavioral adaption to new task demands and where less functional reconfiguration reflects greater rigidity in transitions between rest and task (Bassett et al., 2011; Betzel et al., 2017). Using the latter interpretation of functional reconfiguration, Amico et al. (2020) uncovered a transient network reconfiguration process during the rest period after task using an approach that extracted independent components of FCs (Amico et al., 2017), which was diminished in individuals with FHA (as well as males).

In this work, we examined the relationship of FHA and functional reconfiguration using the correlation distance of tangent-FCs. The results with this novel framework show that functional reconfiguration from task to rest is affected by family history, with those with FHA had less functional reconfiguration. Greater recent drinking behavior (the first principal component of the AUDIT and self-reported drinking) was also associated with reduced functional reconfiguration in the transition from rest to task. These findings indicate that AUD risk factors alter functional reconfiguration both while engaging in and disengaging from the SST.

Specifically, in the model for engaging in the SST, those with lower recent drinking functionally reconfigure more after accounting for age, sex, education, and task performance. Sex and depressive symptoms were also significant predictors. Although it is important to control for potential sex effects in brain function, men are at greater risk of AUD (Flores-Bonilla & Richardson, 2020; Grant et al., 2015) and reconfigured less when engaging in the SST (Figure 6A). Insofar as negative affect/depression figures prominently in AUD, it was unexpected that higher depression scores on the CES-D inventory would relate to greater functional reconfiguration, which might stem from a greater degree of dynamic functional connectivity fluctuations in depression (Lin et al., 2023).

When disengaging from the SST, we observed anticipated FHA effects (i.e., FHA-positive individuals functionally reconfigured less), after controlling for education and CES-D (Figure 6B). Similar to the rest-to-task transition, the CES-D score was again positively related with the task-to-rest reconfiguration. Note that CES-D was included to control for depression symptoms, but for the entire cohort, mean and standard deviation are 6 and 5, respectively, and where only three subjects are above the clinical threshold (16). The score of FHA-positive participants was, however, significantly greater, and even a limited symptom burden might be sufficient to affect the transition between cognitive states.

It has been suggested that better task performance is related to more efficient (smaller) functional reconfiguration (Schultz & Cole, 2016). From that perspective, it could be expected that SSRT (performance measure of the SST) is associated with functional reconfiguration during engaging (and possibly during disengaging). However, SSRT was not significantly associated with functional reconfiguration when engaging or disengaging in our final models (Figure 6). This could be consequence of a limited sample size and/or because SSRT is associated with functional reconfiguration of specific functional circuits rather than of the whole brain.

The results further show that transitions between the task and rest are asymmetric in that the reconfiguration is more prominent during engagement as compared with disengagement (Figure 5B). We evaluated this observation with respect to the choice of fMRI segments to compute the reference matrix. Our results suggest that the magnitudes of functional reconfiguration were consistent for both engaging and disengaging across the three choices of fMRI segments for computing the reference (Supporting Information Figure S1).

Furthermore, when we measure functional reconfiguration from SST to R2.2 instead of R2.1 as the predicted variable, the multilinear regression model results were similar to those displayed in Figure 6B (i.e., the same predictors were significant, there were only minor differences in the explained variance; Supporting Information Figure S2B). Thus, the effects of the FHA group membership on our quantification of functional reconfiguration were not transient but instead spanned the entire rest period after SST.

In contrast to task-to-rest and rest-to-task transitions, AUD risk variables were not significant in within-rest transitions after the SST, consistent with our hypotheses. The only significant variable in the final model was education. This suggests that the within-rest fluctuations of functional connectivity following task engagement were unaffected by the SST. Interestingly, the model for within-rest transition in the first scan (R1.1 to R1.2) was also similar to Figure 6C, with education again as the only significant predictor (Supporting Information Figure S2A).

We performed leave-one-out cross-validation analyses to study the stability of the regression coefficient estimation in the multilinear regression models and the computation of reference matrix, *C*_*ref*_, for tangent space projection. The results in Figures 7 and 8 show that the models presented in Figure 6 were not driven by a particular choice of *C*_*ref*_ and were stable across the 54 leave-one-out models fitted. While these results show high stability on the regression coefficients of the final models within our cohort, a larger sample is required to assess the generalizability of our results.

Finally, we evaluated the effects of regularization parameter on the multilinear regression models. While identification rates were high (>98%) for all tested values, Figure 9 clearly shows that when using correlation distance on tangent-FCs, smaller regularization values best preserve the explanation power of tangent-FCs compared with larger values (≥0.1). This is due to the *shrinking effect* of regularization on the values of functional couplings in tangent-FCs; the higher the regularization value, the smaller the range and variance of functional couplings (Abbas et al., 2021) (Supporting Information Figure S4). The loss of variance leads to the loss of any meaningful differences between functional couplings. Therefore, depending on the dataset, we recommend testing a range of regularization values starting with the smallest that ensure positive definiteness. Note that although the amount of regularization does not affect the fingerprinting accuracy of tangent-FCs when using correlation distance (Abbas et al., 2023), our results show that it greatly influences accuracy when explaining functional reconfiguration (Figure 9).

In this dataset reflective of AUD risk, we showed that functional reconfiguration from rest to SST (engaging) and SST to rest (disengaging) measured using tangent-FCs was associated with AUD risk factors. Recent drinking behavior is associated with engaging functional reconfiguration; those with greater recent drinking behavior showed less functional reconfiguration engaging in SST. On the other hand, disengaging from the SST was affected by the FHA group membership; FHA-positive individuals had less functional reconfiguration while disengaging from the SST. Furthermore, our findings suggest that small values (provided positive definiteness depending on the dataset) preserve the variance of functional couplings, thus preserving the explanation power of tangent-FCs. We aim to expand on the functional reconfiguration framework proposed here by including fMRI designs with the rest period before task within the same scan. Our results suggest that analysis of functional reconfiguration using tangent-FCs is a promising avenue to better understand rest-to-task and task-to-rest brain transitions.

## ACKNOWLEDGMENTS

This work was supported by NIH CTSI CTR EPAR2169, NIH R21 AA029614, NIH R01 AA029607, Indiana Alcohol Research Center P60AA07611, NINDS R01NS126449, and NINDS R01NS112303. This research was also supported in part by Lilly Endowment Inc. through its support for the Indiana University Pervasive Technology Institute and by Shared University Research grants from IBM Inc. to Indiana University. We would like to thank Evgeny Chumin and Andrea Avena-Koenigsberger for their invaluable help in testing of the functional analysis pipeline and image visualization. We greatly appreciate Yu-Chien Wu from the Indiana Institute of Biomedical Imaging Sciences (IIBIS) In-Vivo Imaging Core for assistance with sequence development and testing, as well as MRI technologists Michele Dragoo, Traci Day, and Robert Bryant for their help during imaging sessions. Finally, we greatly appreciate work by all technicians in David Kareken’s laboratory.

## SUPPORTING INFORMATION

Supporting information for this article is available at https://doi.org/10.1162/netn_a_00419.

## AUTHOR CONTRIBUTIONS

Mahdi Moghaddam: Conceptualization; Formal analysis; Investigation; Methodology; Visualization; Writing – original draft; Writing – review & editing. Mario Dzemidzic: Conceptualization; Data curation; Formal analysis; Funding acquisition; Investigation; Methodology; Supervision; Validation; Writing – original draft. Daniel Guerrero: Formal analysis; Methodology; Writing – review & editing. Mintao Liu: Conceptualization; Formal analysis; Methodology; Writing – review & editing. Jonathan Alessi: Conceptualization; Methodology. Martin H. Plawecki: Conceptualization; Data curation; Investigation; Writing – review & editing. Jaroslaw Harezlak: Conceptualization; Formal analysis; Funding acquisition; Investigation; Methodology; Writing – review & editing. David Kareken: Conceptualization; Data curation; Formal analysis; Funding acquisition; Investigation; Methodology; Resources; Supervision; Validation; Visualization; Writing – original draft; Writing – review & editing. Joaquín Goñi: Conceptualization; Formal analysis; Funding acquisition; Investigation; Methodology; Supervision; Visualization; Writing – original draft; Writing – review & editing.

## FUNDING INFORMATION

Joaquín Goñi, National Institutes of Health (US), Award ID: CTSI CTR EPAR2169. David Kareken, National Institutes of Health (US), Award ID: R21 AA029614. David Kareken, National Institutes of Health (US), Award ID: R01 AA029607. David Kareken, National Institutes of Health (US), Award ID: P60AA07611. Jaroslaw Harezlak, National Institutes of Health (US), Award ID: R01NS126449. Jaroslaw Harezlak, National Institutes of Health (US), Award ID: R01NS112303.

## Supplementary Material



## TECHNICAL TERMS

Functional magnetic resonance imaging (fMRI): A noninvasive imaging method that detects changes in levels of blood oxygenation, which is associated with neuronal activation.
Functional connectome (FC) matrix: A symmetric matrix whose elements represent the statistical dependencies between time series of brain regions (in this study, pairwise Pearson’s correlation). The time series are obtained from functional magnetic resonance imaging (fMRI). Here, an FC is a correlation matrix that includes all the pairwise functional couplings between brain regions.
Family history of alcohol use disorder (FHA): A participant is considered FHA positive if they have at least one first-degree relative with a history of alcohol use disorder (AUD).
Tangent space: A Euclidean space formed by the collection of vectors that are the derivatives of the curves crossing a reference matrix on the SPD manifold.
Tangent space projection: A Riemannian geometry technique where the matrices on the SPD manifold are mapped to a tangent space of a reference matrix on the manifold.
Tangent-FCs: The resulting vector of tangent space projection of FCs.
Reference matrix: A matrix that belongs to the SPD manifold that is used to perform a tangent space projection. This matrix is where the tangent space and the SPD manifold intersect.
SPD manifold: The non-Euclidean space of symmetric positive definite matrices.
Engaging functional reconfiguration: The amount of change in functional connectivity of an individual in a rest-to-task transition, quantified by the correlation distance of their corresponding FCs.
Disengaging functional reconfiguration: The amount of change in functional connectivity of an individual in a task-to-rest transition, quantified by the correlation distance of their corresponding tangent-FCs.
Stop signal task (SST): A cognitively engaging task consisting of Go and Stop trials. Participants are instructed to respond as quickly and accurately as possible to a Go signal by pressing a right or left button on an MRI-compatible button box and inhibit their response during Stop trials signaled by a red up-pointing arrow immediately following a Go stimulus.
